# Screening and Identification of Key Common and Specific Genes and Their Prognostic Roles in Different Molecular Subtypes of Breast Cancer

**DOI:** 10.3389/fmolb.2021.619110

**Published:** 2021-02-11

**Authors:** Na Sun, Pingping Gao, Yanling Li, Zexuan Yan, Zaihui Peng, Yi Zhang, Fei Han, Xiaowei Qi

**Affiliations:** ^1^ Department of Breast and Thyroid Surgery, Southwest Hospital, Army Medical University, Chongqing, China; ^2^ Institute of Pathology and Southwest Cancer Center, Key Laboratory of the Ministry of Education, Southwest Hospital, Army Medical University, Chongqing, China; ^3^ Institute of Toxicology, College of Preventive Medicine, Army Medical University, Chongqing, China

**Keywords:** breast cancer, molecular subtypes, prognosis, biomarkers, diagnosis-breast cancer

## Abstract

Breast cancer is one of the most common cancers. Although the present molecular classification improves the treatment effect and prognosis of breast cancer, the heterogeneity of the molecular subtype remains very complex, and the applicability and effectiveness of treatment methods are still limited leading to poorer patient prognosis than expected. Further identification of more refined molecular typing based on gene expression profile will yield better understanding of the heterogeneity, improving treatment effects and prolonging prognosis of patients. Here, we downloaded the mRNA expression profiles and corresponding clinical data of patients with breast cancer from public databases and performed typical molecular typing using PAM50 (Prediction Analysis of Microarray 50) method. Comparative analyses were performed to screen the common and specific differentially expressed genes (DEGs) between cancer and corresponding para-cancerous tissues in each breast cancer subtype. The GO and KEGG analyses of the DEGs were performed to enrich the common and specific functional progress and signaling pathway involved in breast cancer subtypes. A total of 38 key common and specific DEGs were identified and selected based on the validated results, GO/KEGG enrichments, and the priority of expression, including four common DEGs and 34 specific DEGs in different subtypes. The prognostic value of these key common and specific DEGs was further analyzed to obtain useful potential markers in clinic. Finally, the potential roles and the specific prognostic values of the common and specific DEGs were speculated and summarized in total breast cancer and different subtype breast cancer based on the results of these analyses. The findings of our study provide the basis of more refined molecular typing of breast cancer, potential new therapeutic targets and prognostic markers for different breast cancer subtypes

## Introduction

Breast cancer is one of the few tumor types with good molecular classification and targeted therapies (Barry et al., 2010; Prat et al., 2015). However, due to its complex heterogeneity, the current treatment effects and patient’s prognosis are still not very satisfactory. An increasing number of researchers have begun to focus on the subdivision of breast cancer subtypes for individualizing treatment, which may be the main means of fundamentally improving treatment effects and prolonging patient prognosis. The rapid development of molecular biology has prompted the shift of breast cancer from pure anatomical and pathological classification to new classification and fine classification based on molecular standards. Breast cancer can be divided into molecular subtypes with unique clinical features and identifiable gene expression features (Prat et al., 2015). PAM50 (Prediction Analysis of Microarray 50) gene signatures are a second-generation multi-gene expression assay used for quantifying the mRNA expression of 50 genes, including *ER*, *PR*, and *Her2*. It is currently recognized in the industry as a molecular subtype classification method for breast cancer. The PAM50 gene signature method was proposed by Parker et al. to evolve from the initial intrinsic subtype, providing 50 gene signatures for subtype assignment (Parker et al., 2009). According to the PAM50 method, breast cancer can be divided into the following five molecular subtypes: Basal-like, LumA, LumB, Her2, and Normal-like (Perou et al., 2000). At present, comprehensive treatment based on molecular typing, including surgery, radiotherapy, chemotherapy, endocrine therapy and targeted therapy, can significantly improve the therapeutic effect, including the overall survival (OS) rate and progression-free survival rate (Edenfield et al., 2017). Although the PAM50 classification improves the treatment effect and prognosis of breast cancer, the problems remain. As the heterogeneity of the same molecular subtype remains very complex, the applicability and effectiveness of treatment methods are still very limited, resulting in poorer patient prognosis than expected (Sotiriou et al., 2003). Moreover, the differences in the molecular characteristics and pathways among patients with Basal-like, Her2, LumA, LumB, and Normal-like breast cancer subtypes are still not well understood.

To choose more suitable treatment methods for different breast cancer molecular subtypes, better and deeper understanding of the similarities and differences of the patients’ biological and molecular pathways are required. The exploration of abnormally expressed genes during breast cancer development is essential for in-depth understanding of the biological functions, molecular pathways, and possible mechanisms involved. However, the different genetic backgrounds and living environments of different populations complicate the identification of the common tumor-related genes associated with breast cancer. Transcriptome sequencing and bioinformatics analysis can evaluate the whole cell tissue process effectively (Martin and Wang, 2011). Whole-transcriptome analysis can reveal differentially expressed genes (DEGs) in relevant tissues (e.g., breast cancer tumor tissues and paracancerous breast tissues), elucidate their cellular mechanisms and development processes, and can yield potentially more valuable therapeutic, diagnostic, and prognostic targets (Sørlie et al., 2001).

Elucidating the potential biological and molecular pathways of patients with different breast cancer molecular subtypes is necessary for selecting effective treatment modalities and for improving treatment efficacy and patient’s prognosis. The present study was aimed to identify the DEGs between the cancer tissues and paracancerous tissues (cancer-adjacent normal tissue) from the different breast cancer subtypes, screen key common and specific differential molecules and signaling pathways, and analyze the clinical application value of these key molecules. At last, the possibility of these key differential molecules as potential diagnostic and prognostic markers and novel therapeutic targets for different breast cancer subtypes was evaluated through comprehensive analyses.

## Materials and Methods

### Data Download

The Cancer Genome Atlas (TCGA) breast cancer (BRCA-RNAseq) data were downloaded from the UCSC Xena database, and included 1,091 cancer tissue samples. Using the genefu software package of R 4.0 (Gendoo et al., 2016), the samples underwent molecular classification using the PAM50 method. The Basal-like (*N* = 190), Her2 (*N* = 82), LumA (*N* = 564), LumB (*N* = 215), and Normal-like (*N* = 40) molecular subtypes were compared, and common and specific DEGs in the subtypes were screened. Similarly, METABRIC data were downloaded, and the Basal-like (*N* = 199), Her2 (*N* = 220), LumA (*N* = 680), LumB (*N* = 461), and Normal-like (*N* = 140) subtypes were compared according to PAM50 subtype classification annotated by METABRIC data. The subtypes’ common and specific DEGs were screened, and the results of screening with TCGA analysis data were mutually verified to obtain the candidate common and specific DEGs.

### DEG Screening Analysis

Cancer samples from patients with the five breast cancer subtypes were matched to the corresponding paracancerous sample data; genes without corresponding gene names were deleted, and data <0 were corrected. DEGs were determined using the limma package in R 4.0. The screening criteria were | log two FC (fold change) | >1.5 and *p* < 0.05; gene_ID was converted into ENTREZID and gene symbol, and genes without the corresponding ENTREZID and gene symbols were discarded to obtain their respective DEGs. Comparing the subtypes DEG profiles, a Venn diagram was used to determine the common and specific DEGs among the subtypes. The Venn diagram was created using jvenn (Bardou et al., 2014). The expression trends of the subtypes’ shared and specific DEGs were verified using METABRIC data and Breast Cancer Gene-Expression Miner v4.5 (bc-GenExMiner v4.5, http://bcgenex.centregauducheau.fr/BC-GEM/GEM-requete.php). Limma package has a command “Normalize” in built, and the RNAseq-counts data was normalized before the difference analyses.

### Functional Analysis of DEGs

Gene ontology (GO) and Kyoto Encyclopedia of Genes and Genomes (KEGG) pathway enrichment of DEGs was performed by the clusterProfiler package (Yu et al., 2012) and GOplot package in R 4.0, and the conversion results were visualized. When *p* < 0.05, the GO and KEGG pathway was identified as significantly enriched. The top 10 GO terms and KEGG enrichment pathways were mapped using the ggplot2 packages in R 4.0.

### Survival Analysis

Survival analysis was performed by the Kaplan-Meier plotter online tool (www.kmplot.com) (Györffy et al., 2010).The cut-off value was set to select the best cut-off value automatically, and all data sets within the website were selected for analysis according to the Basal-like, Her2, LumA, and LumB subtypes. As the Kaplan-Meier plotter does not have Normal-like subtype grouping data, we screened patients with the Normal-like subtype and the corresponding prognostic data from TCGA using GraphPad Prism 5.

## Results

### DEG Screening

**FIGURE 1:**
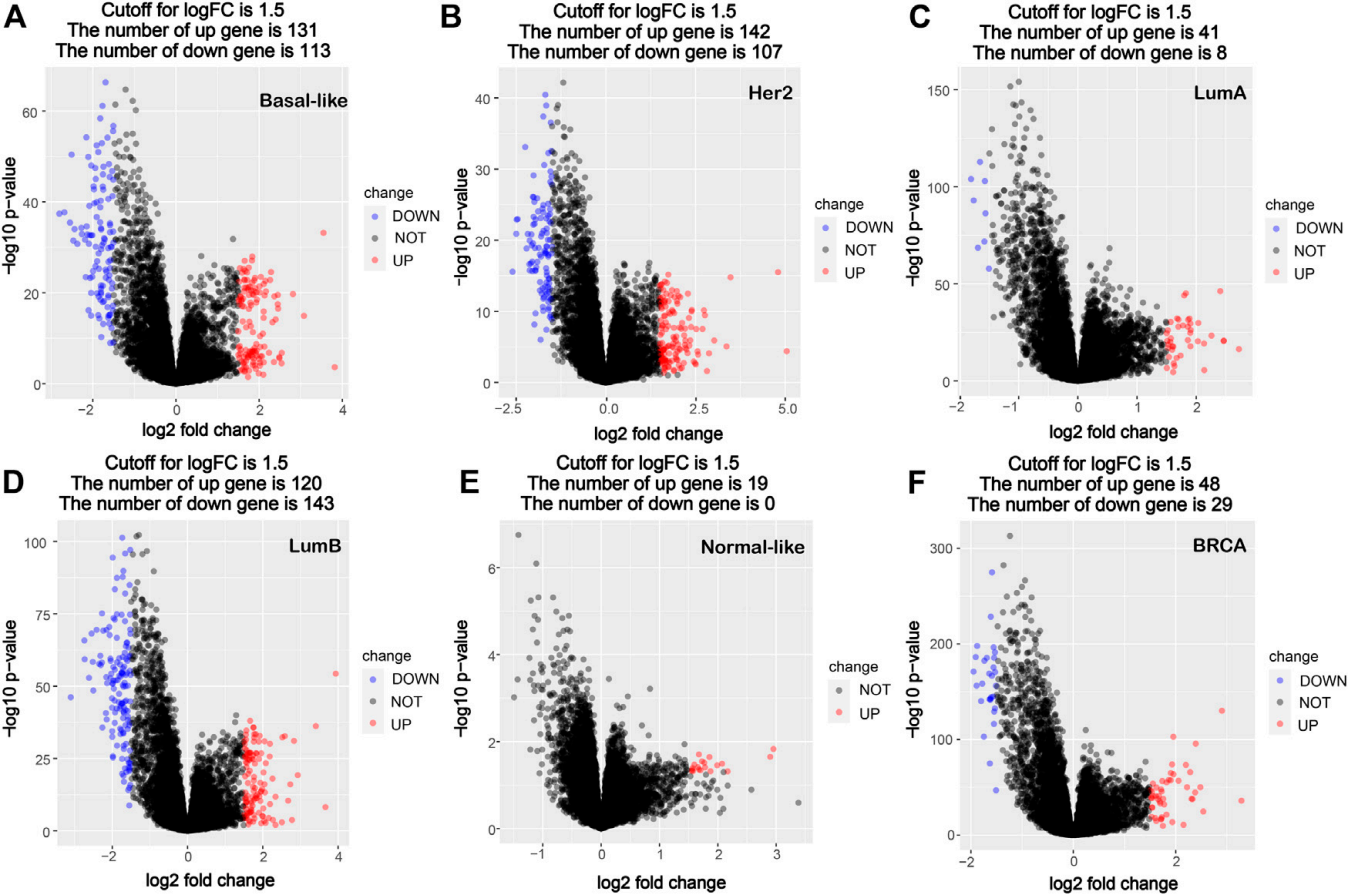
Volcano map of DEGs between cancer tissues and paracancerous tissues in breast cancer molecular subtypes and all breast cancer. **(A)** Basal-like; **(B)** Her2; **(C)** LumA; **(D)** LumB; **(E)** Normal-like; **(F)** BRCA (all breast cancer). Red dots denote upregulated genes screened based on log two FC > 1.5 (*p* < 0.05); blue dots denote downregulated genes screened based on log two FC < -1.5 (*p* < 0.05); black dots denote genes with no significant difference (*p* < 0.05). *p* < 0.05, FC, fold change.

Using the PAM50 method, the breast cancer samples (1,091 cases) in TCGA database were divided into five molecular subtypes: Basal-like (190 cases), Her2 (73 cases), LumA (564 cases), LumB (215 cases) and Normal-like (40 cases). A comparative analysis of gene expression was performed between cancer tissues and corresponding paracancerous tissues in all breast cancer and different subtypes’ breast cancer, and the significantly differentially expressed genes were screened out. Compared with the corresponding paracancerous tissues, 131 high-expression genes and 113 low-expression genes were screened out in the Basal-like subtype, 142 high-expression genes and 107 low-expression genes were screened out in the Her2 subtype, 41 high-expression genes and eight low-expression genes were screened out in the LumA subtype, 120 high-expression genes and 143 low-expression genes were screened out in the LumB subtype, and 19 high-expression genes and no significant low-expression genes were screened out in the Normal-like subtype, whereas 48 high-expression genes and 29 low-expression genes were screened out in all breast cancers (Figure 1). The data of comparative analysis suggest that the changes in the molecular characteristics in the breast cancer subtypes may be quite different from that in breast cancer as a whole.

**FIGURE 2:**
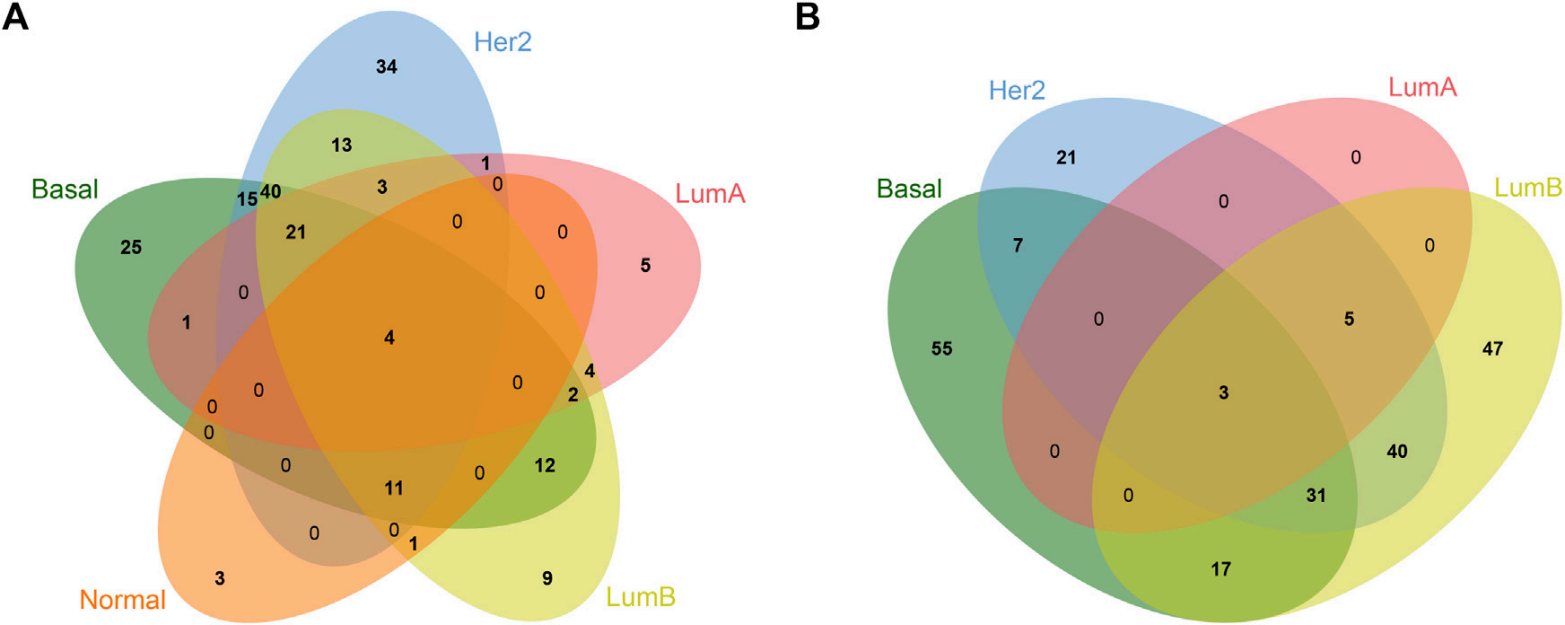
Venn diagram of common and specific DEGs in different breast cancer subtypes. **(A)** Upregulated DEGs; **(B)** Downregulated DEGs.

To analyze the common and specific DEGs in the five subtypes, we performed the intersection on the Venn diagram. There were four common DEGs in the five subtypes. There were 80 specific DEGs for the basal-like subtype, 55 specific DEGs for the Her2 subtype, five specific DEGs for the LumA subtype, 56 specific DEGs for the LumB subtype, and three specific DEGs for the Normal-like subtype (Table 1; Figure 2). Supplementary Table S1 shows the details of the common DEGs in the five subtypes and the specific DEGs for each subtype.

**TABLE 1:** Number of common and specific DEGs in the breast cancer subtypes.

PAM50 subtype	Common	Basal-like	Her2	LumA	LumB	Normal-like
UP	4	25	34	5	9	3
DOWN	0	55	21	0	47	0

### GO Analysis of the DEGs

**FIGURE 3:**
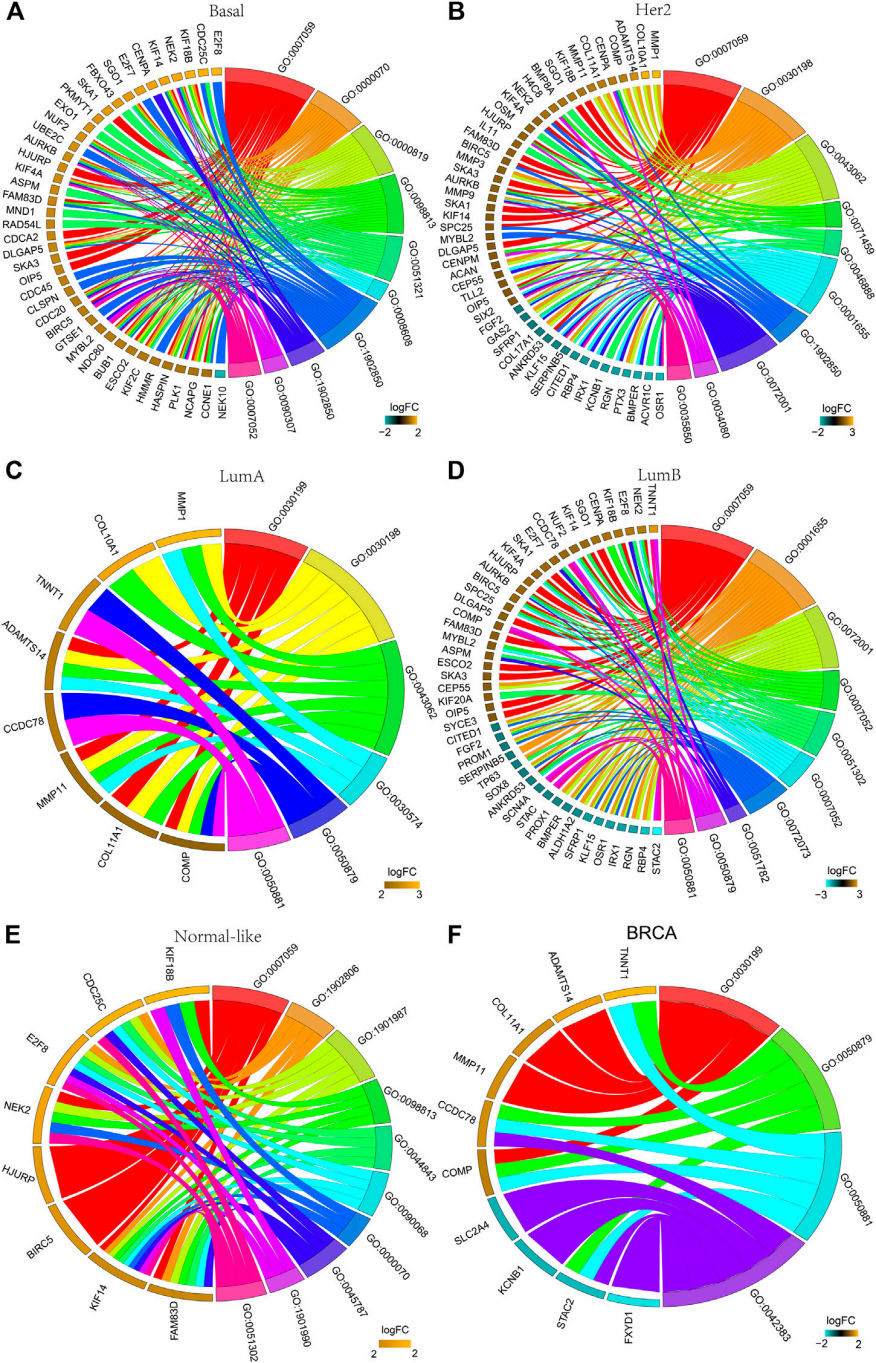
GO analysis of the DEGs. The chord plot displays the relationship between DEG and GO terms, representing the names of DEGs in the top enriched 10 GO terms. **(A)** Basal-like; **(B)** Her2; **(C)** LumA; **(D)** LumB; **(E)** Normal-like; **(F)** BRCA (all breast cancer). GO:0007059: chromosome segregation; GO:0000070: mitotic sister chromatid segregation; GO:0000819: sister chromatid segregation; GO:0098813: nuclear chromosome segregation; GO:0051321: meiotic cell cycle; GO:0008608: attachment of spindle microtubules to kinetochore; GO:1901987: regulation of cell cycle phase transition; GO:1902850: microtubule cytoskeleton organization involved in mitosis; GO:0090307: mitotic spindle assembly; GO:0007052: mitotic spindle organization; GO:0030198: ECM organization; GO:0043062: extracellular structure organization; GO:0071459: protein localization to chromosome, centromeric region; GO:0046888: negative regulation of hormone secretion; GO:0001655: urogenital system development; GO:0072001: renal system development: GO:0034080: CENP-A containing nucleosome assembly; GO:0035850: epithelial cell differentiation involved in kidney development; GO:0030199: collagen fibril organization; GO:0030574: collagen catabolic process; GO:0050879: multicellular organismal movement; GO:0050881: musculoskeletal movement; GO:0051302: regulation of cell division; GO:0072073: kidney epithelium development; GO:0051782: negative regulation of cell division; GO:1902806: regulation of cell cycle G1/S phase transition; GO:0044843: cell cycle G1/S phase transition; GO:0090068: positive regulation of cell; GO:0045787: positive regulation of cell cycle; GO:1901990: regulation of mitotic cell cycle phase transition; GO:0042383: sarcolemma.

The functions of the DEGs in all breast cancers and each subtype breast cancer were predicted using GO analysis. Figure 3 shows the top 10 enriched GO entries of all breast cancers and each subtype breast cancer, and the detailed results of all enriched GO entries are shown in Supplementary Tables S2–S7. Comparative analysis of the subtypes’ GO entries showed that a total of 123 GO entries were enriched for the DEGs in the basal-like subtype, and 61 of these GO entries were specifically enriched which were mainly concentrated in “mitotic,” “cell cycle,” and “oxidoreductase activity.” A total of 39 GO entries were enriched for the DEGs in the Her2 subtype, and 10 of these GO entries were specifically enriched which were mainly concentrated in “nucleosome,” “cell differentiation” and “RAGE receptor binding.” A total of six GO entries were enriched for the DEGs in the LumA subtype, whereas there was no specifically enriched GO entry. A total of 50 GO entries were enriched for the DEGs in the LumB subtype, and 13 of these 50 GO entries were specifically enriched that were mainly concentrated in “kidney development” and “chloride channel complex.” A total of 86 GO entries were enriched for the DEGs in the normal-like subtype, and 41 of these GO entries were specifically enriched that were mainly concentrated in “mitotic DNA damage checkpoint,” and “DNA damage response.”

There were no co-enriched GO entries for the DEGs among the five subtypes. There were 13 co-enriched GO entries for the DEGs among the Basal-like, Her2, LumB and Normal-like subtypes, mainly at “chromosome” and “mitosis.” There was one GO entry for co-enriched of the DEGs in Basal-like, Her2 and LumA subtypes: “collagen fibril organization.” There were four co-enriched GO entries for the DEGs in the Basal-like, Her2 and LumB subtypes, mainly at “CENP-A containing nucleosome assembly,” “protein localization to chromosome” and “CENP-A containing chromatin organization.” There were two co-enriched GO entries for the DEGs in the LumA and LumB subtypes, mainly at “multicellular organismal movement” and “musculoskeletal movement.”

### KEGG Pathway Analysis of the DEGs

**FIGURE 4:**
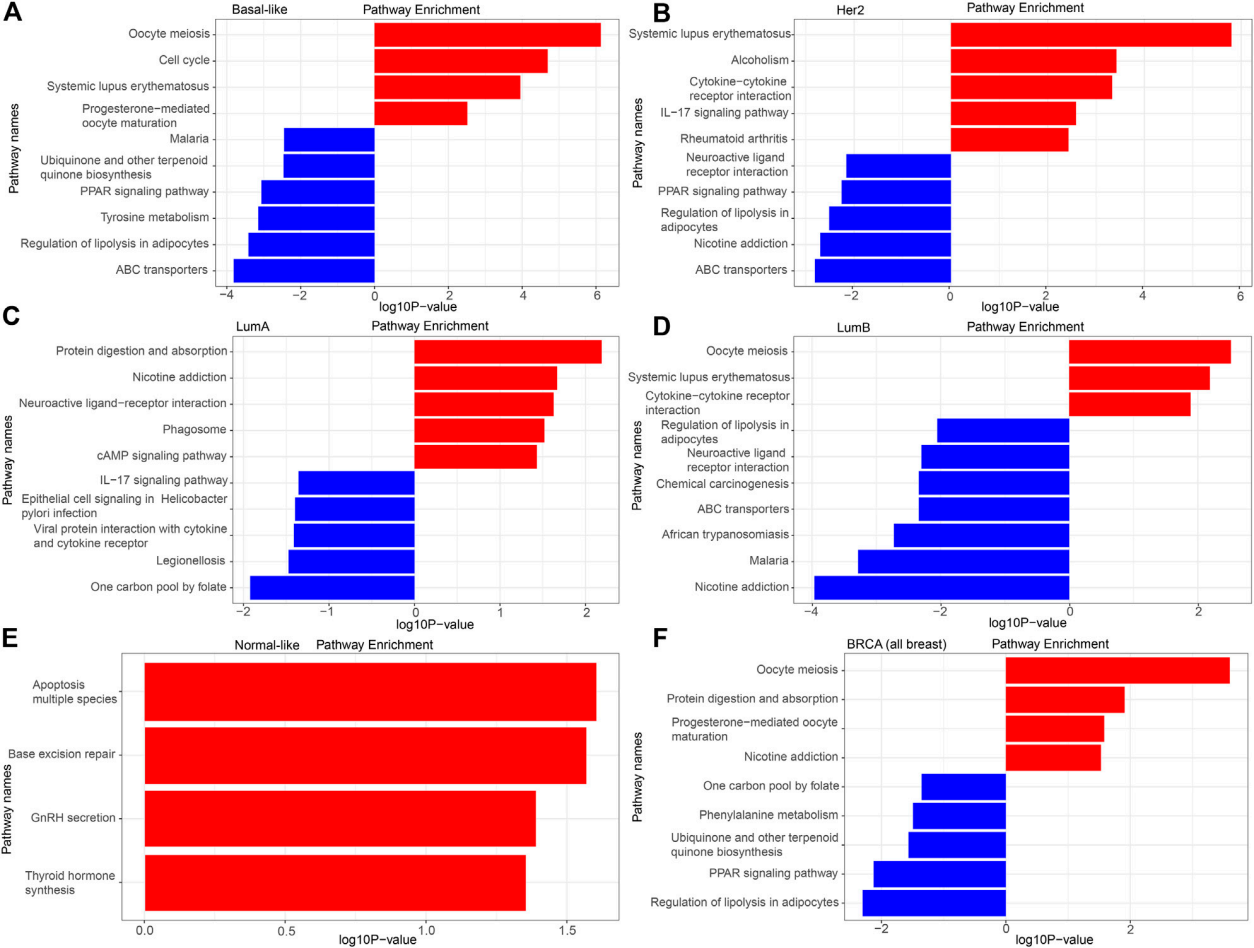
KEGG analysis of the DEGs. **(A)** Basal-like; **(B)** Her2; **(C)** LumA; **(D)** LumB; **(E)** Normal-like; **(F)** BRCA (all breast cancer).

The detailed KEGG pathway enrichment results are shown in Supplementary Tables S8–S13, and the top 10 KEGG pathways enriched of all breast cancer and each subtype were represented in Figure 4. In the Basal-like subtype, the DEGs were enriched in 22 KEGG pathways, and eight of these pathways were specifically enriched mainly focusing on “ubiquinone and other terpenoid-quinone biosynthesis,” “maturation” and “amino acid metabolism.” In the Her2 subtype, the DEGs were enriched in 16 KEGG pathways, and four of these 16 pathways were specifically enriched mainly focusing on “bladder cancer” and “amino acid metabolism.” In the LumA subtype, the DEGs were enriched in 11 KEGG pathways, and five of these pathways were specifically enriched mainly focusing on “epithelial cell signaling in infection,” “one carbon pool by folate,” “phagosome” and “protein interaction with cytokine.” In the LumB subtype, the DEGs were enriched in 24 KEGG pathways, and 10 of these 24 pathways were specifically enriched mainly focusing on “chemical carcinogenesis,” “insulin resistance” and “drug metabolism.”

There was no pathway for co-enrichment of the DEGs in the five subtypes, but there was one pathway for co-enrichment of the DEGs in the Basal-like, Her2, LumA, and LumB subtypes: “neuroactive ligand-receptor interaction.” There was also one pathway for co-enrichment of the DEGs in the basal-like, Her2 and LumA subtypes: “rheumatoid arthritis.” There were six pathways for co-enrichment of the DEGs in the Basal-like, Her2 and LumB subtypes focusing on “alcoholism,” “ABC transporters,” “PPAR signaling pathway,” “regulation of lipolysis in adipocytes,” “oocyte meiosis” and “systemic lupus erythematosus.” There was one pathway for co-enrichment of the DEGs in the basal-like, LumA and LumB subtypes: “protein digestion and absorption.” There were two pathways for co-enrichment of the DEGs in the Her2, LumA and LumB subtypes: “nicotine addiction” and “IL-17 signaling pathway.” There was one pathway for co-enrichment of the DEGs in the basal-like and Her2 subtypes: “cysteine and methionine metabolism.” There was one pathway for co-enrichment of the DEGs in the basal-like and LumA subtypes: “cAMP signaling pathway.” There were three pathways for co-enrichment of the DEGs in the basal-like and LumB subtypes: “cell cycle,” “malaria” and “African trypanosomiasis.” There was one pathway for co-enrichment of the DEGs in the Her2 and LumB subtypes: “cytokine-cytokine receptor interaction.”

### Screening and Validation of the Key Common and Specific Genes From the DEGs

To analyze the crucial common and specific genes in the subtypes, we first validated the potential DEGs using the METABRIC database and bc-GenExMiner v4.5. The DEGs with consistent results in the different databases were retained. After verification, four key common DEGs among the subtypes were identified: *NEIL3*, *CDC25C*, *NEK2* and *HCN2*, and their expression were significantly upregulated in all five subtypes. The specific DEGs were 61 genes (21 upregulated and 40 downregulated) for Basal-like subtype, 34 genes (19 upregulated and 15 downregulated) for Her2 subtype, two genes (both upregulated) for LumA subtype, 36 genes (3 upregulated and 33 downregulated) for LumB subtype, and two genes (both upregulated) for Normal-like subtype (Table 2). To facilitate further analysis, we further selected the key specific DEGs for each subtype. As just a few validated DEGs were found in the LumA and Normal-like subtypes, these specific DEGs were considered to be the key genes by default. There were many specific DEGs in the Basal-like, Her2 and LumB subtypes, and the top 10 were selected as the key specific DEG for each subtype by combining the specific GO and KEGG enrichment results and the priority of the changed expression. The selected key specific genes were *MISP* and *SMIM22* for LumA subtype, *IDH1*-*AS1* and *TMEM233* for Normal-like subtype, *MCM10*, *HPDL*, *SOX11*, *PLK1*, *BUB1*, *DYNLRB2*, *OGN*, *COL4A6*, *AGTR1,* and *ADRB2* for Basal-like subtype, *SPOCD1*, *IL21R*, *JPH3*, *SAMD11*, *IFI30*, *ATRNL1*, *TNNI3K*, *PI15*, *FAM189A2,* and *MYZAP* for Her2 subtype, and *CNTD2*, *NEURL1*, *SYCE3*, *STAC2*, *PPP1R1A*, *HRCT1*, *AKR1C2*, *IL6*, *FREM1,* and *HOXA4* for LumB subtype.

**TABLE 2:** Genes with consistent expression with TCGA screening results were verified *via* the METABRIC database and BC-GenExMiner v4.5.

PAM50 subtype	Up gene name	Down gene name
Basal-Her2-LumA-LumB-normal-like	NEIL3/CDC25C/NEK2/HCN2	—
Basal	SLCO5A1/GPR19/LCTL/MCM10/HPDL/**SOX11**/RAD54L/CDCA2/MSLN/GTSE1/NDC80/BUB1/COL22A1/KIF2C/PLK1/FOXM1/CDCA3/NCAPG/AUNIP/**MIR4292**/CCNE1	**LINC00504**/TFF3/CYP4B1/TTC36/DYNLRB2/**OGN**/ACSM5/MS4A2/**MIR4697HG**/C16orf89/PTPRT/MYRIP/CAPN8/AGTR1/PFKFB1/PGR/COL4A6/ABCC11/DHRS2/**NDNF**/PLIN5/FER1L5/AZGP1P1/GRIK3/NEK10/RANBP3L/**FAM198B-AS1**/GFRA1/SOWAHA/CLEC3B/**LRRN3**/**FAM162B**/**AK5**/IQUB/SEMA3E/**ADRB2**/GLDN/DACH1/LIPE/LOC105371730
Her2	**LOC105372233**/SPOCD1/**IL21R**/JPH3/SAMD11/**IFI30**/**JSRP1**/**ANGPTL6**/**IL4I1**/VSTM2L/**MMP3**/**HES6**/**S100A8**/ASCL2/TCHH/NXPH4/LBX2/**LOC105371849/SIX2**	**LINC00173**/**ATRNL1**/**TNNI3K**/PI15/FAM189A2/**LOC100505635**/MYZAP/**SLC4A4**/LRP2/NAT8L/**OXTR**/SLC25A27/**FGF14-AS2**/**LINC00639**/TRMT9B
LumA	**MISP**/**SMIM22**	—
LumB	**CNTD2**/**NEURL1/SYCE3**	STAC2/PPP1R1A/HRCT1/AKR1C2/IL6/FREM1/HOXA4/TMOD1/PTGS2/PTGS2/FAM107A/IL33/TRIM29/TMEM220-AS1/PROX1/GFRA2/SLC28A3/HCAR2/PPARGC1A/**GNG12-AS1**/TDRD10/SOX8/TP63/TTYH1/PTH2R/SLC2A12/KCNA6/PROM1/KRT17/C2orf88/PAMR1/PPP1R14A/BCL11A
Normal-like	**IDH1-AS1**/**TMEM233**	—

(Genes annotated in **yellow** are those for which validation results were discordant or no data were available in the METABRIC database and BC genexminer v4.5).

To validate the accuracy of our expression analysis results, we downloaded some independent BRCA (non-TCGA) data from the GEO database and classified the samples of these BRCA database into different subtypes according to PMA50, and finally selected the GSE65216 dataset as an independent validation to validate the key common and specific DEGs because it is the largest sample size of different subtypes. The validation data show that the selected key specific DEGs are highly consistent with TCGA data, and the consistency is 100% for LumA subtype, 100% for Basal-like subtype, 60% for Her2 subtype (the consistent DEGs are *SAMD11*, *IFI30*, *ATRNL1*, *PI15*, *FAM189A2,* and *MYZAP*), 60% for LumB subtype (the consistent DEGs are *STAC2*, *PPP1R1A*, *HRCT1*, *AKR1C2*, *FREM1,* and *HOXA4*), whereas the common DEGs seem to be very different in the independent BRCA sanples of GSE65216 database (Table 3; Supplementary Figures S1–S4). As no samples of Normal-like subtype were found in the GSE65216 dataset, the selected genes were not validated in Normal-like subtypes.

**TABLE 3:** The key common and specific DEGs selected were verified in the GSE65216 dataset.

**Basal-like**		
Gene name	TCGA	GSE65216
NEIL3	Up	No change
HCN2	Up	Down
NEK2	Up	Up
CDC25C	Up	Up
SOX11	Up	Up
PLK1	Up	Up
BUB1	Up	Up
OGN	Down	Down
AGTR1	Down	Down
COL4A6	Down	Down
ADRB2	Down	Down
DYNLRB2	Down	Down
HPDL	Up	Up
MCM10	Up	Up
**Her2**		
**Gene name**	**TCGA**	**GSE65216**
NEIL3	Up	Down
HCN2	Up	Down
NEK2	Up	No change
CDC25C	Up	Down
ATRNL1	Down	Down
FAM189A2	Down	Down
IFI30	Up	Up
IL21R	Up	No change
JPH3	Up	Down
MYZAP	Down	Down
PI15	Down	Down
SAMD11	Up	Up
SPOCD1	Up	No change
TNNI3K	Down	No change
**LumA**		
**Gene name**	**TCGA**	**GSE65216**
NEIL3	Up	No change
HCN2	Up	Down
NEK2	Up	No change
CDC25C	Up	No change
MISP	Up	Up
SMIM2	Up	Up
**LumB**		
**Gene name**	**TCGA**	**GSE65216**
NEIL3	Up	No change
HCN2	Up	Down
NEK2	Up	Down
CDC25C	Up	Down
AKR1C2	Down	Down
CNTD2	Up	No change
FREM1	Down	Down
HOXA4	Down	Down
HRCT1	Down	Down
IL6	Down	No change
NEURL1	Up	Down
PPP1R1A	Down	Down
STAC2	Down	Down
SYCE3	Up	No change

### Prognostic Value of the Selected Key Common and Specific DEGs

To determine the importance and clinical value of the selected key common and specific DEGs, we examined the relationship between those genes and the recurrence-free survival (RFS) and OS of patients with breast cancer by Kaplan-Meier analysis. For the four key common DEGs, high expression of *NEIL3* predicted high RFS (*p* < 0.05) and OS (*p* < 0.05), high expression of *NEK2* predicted low RFS (*p* < 0.05) but high OS (*p* < 0.05), and low expression of *CDC25C* only predicted low OS (*p* < 0.05) in the Basal-like subtype (Table 4; Supplementary Figures S5A). High expression of *NEIL3* predicted high RFS (*p* < 0.05) in the Her2 subtype (Table 4; Supplementary Figure S5B). Low expression of *NEIL3*, *CDC25C* and *NEK2* predicted high RFS (*p* < 0.05) and OS (*p* < 0.05), respectively, while low expression of *HCN2* only predicted high OS (*p* < 0.05) in the LumA subtype (Table 4; Supplementary Figure S5C). Low expression of *CDC25C* predicted high OS (*p* < 0.05), high expression of *NEIL3* and *HCN2* predicted high RFS (*p* < 0.05) in the LumB subtype (Table 4; Supplementary Figure S5D). Low expression of *NEIL3* predicted high OS (*p* < 0.05), while low expression of NEK2 predicted high RFS (*p* < 0.05) and OS (*p* < 0.05) respectively, while in the LumB subtype (Table 4; Supplementary Figure S5D). In all breast cancer, low expression of *NEIL3*, *CDC25C* and *NEK2* predicted high RFS (*p* < 0.05) respectively, while high expression of *HCN2* predicted high RFS (*p* < 0.05), and low expression of *NEIL3*, *CDC25C*, *NEK2* and *HCN2* predicted high OS respectively (*p* < 0.05) (Table 4; Supplementary Figure S5E).

**TABLE 4:** Prognostic value of the key common DEGs in total and different subtype breast cancers.

**Basal**	**RFS**		**OS**	
Gene	HR (95% CI for HR)	P	HR (95% CI for HR)	P
NEIL3	0.68 (0.53–0.88)	0.003	0.57 (0.34–0.93)	0.022
CDC25C	0.91 (0.7–1.17)	0.45	0.5 (0.3–0.83)	0.0063
NEK2	1.35 (1.03–1.79)	0.031	0.55 (0.34–0.9)	0.016
HCN2	0.77 (0.58–1.03)	0.074	1.36 (0.83–2.23)	0.23
**HER2**	**RFS**		**OS**	
NEIL3	0.59 (0.4–0.86)	0.006	1.38 (0.71–2.7)	0.34
CDC25C	0.7 (0.47–1.04)	0.078	0.56 (0.26–1.23)	0.14
NEK2	1.34 (0.91–1.97)	0.13	0.55 (0.23–1.32)	0.17
HCN2	1.54 (0.98–2.42)	0.057	1.94 (0.91–4.11)	0.079
**Lum A**	**RFS**		**OS**	
NEIL3	1.29 (1.08–1.54)	0.0041	1.79 (1.24–2.59)	0.0017
CDC25C	1.64 (1.37–1.96)	6e-08	2 (1.4–2.85)	9.2e-05
NEK2	2.2 (1.85–2.6)	<1e-16	2.5 (1.75–3.57)	1.6e-07
HCN2	0.86 (0.72–1.02)	0.09	1.5 (1.03–2.19)	0.034
**Lum B**	**RFS**		**OS**	
NEIL3	0.81 (0.67–0.98)	0.03	1.73 (1.11–2.69)	0.014
CDC25C	1.19 (0.99–1.45)	0.068	1.49 (1.03–2.15)	0.035
NEK2	1.71 (1.37–2.14)	1.7e-06	1.86 (1.17–2.96)	0.0082
HCN2	0.74 (0.6–0.91)	0.0047	1.36 (0.94–1.97)	0.1
**BRCA**	**RFS**		**OS**	
NEIL3	1.12 (1.07–1.38)	0.0022	1.63 (1.27–2.11)	0.00014
CDC25C	1.45 (1.3–1.61)	2.6e-11	1.54 (1.24–1.91)	8.9e-05
NEK2	1.91 (1.7–2.14)	<1e-16	2.08 (1.62–2.67)	4.8e-09
HCN2	0.83 (0.74–0.94)	0.0033	1.39 (1.1–1.77)	0.0064

For the key specific DEGs in the LumA subtype, *SMIM22* was significantly overexpressed, but the high expression of *SMIM22* was not associated with prognosis (OS and RFS) of patients. As the Kaplan-Meier plotter does not contain information on the *MISP* gene, the prognostic analysis of *MISP* was not performed.

For the key specific DEGs in Normal-like subtype, the prognostic analysis of *IDH1*-*AS1* and *TMEM233* was performed using data from TCGA database, which was not meaningful due to the small number of breast cancer samples from this subtype in TCGA database. Therefore, this part of the results is not shown.

For the key specific DEGs in the Basal-like subtype, low *SOX11* mRNA expression predicted high RFS and OS (*p* < 0.05) (Table 5; Supplementary Figure S6). High expression of *BUB1*, *OGN*, *COL4A6*, *AGTR1,* and *ADRB2* mRNA predicted high RFS respectively (*p* <0.05), and high expression of *BUB1* and *PLK1* mRNA predicted high OS respectively (*p* < 0.05) (Table 5; Supplementary Figure S6). The expression of *MCM10*, *HPDL,* and *DYNLRB2* had no significant correlation with RFS and OS of patients (*p* > 0.05).

**TABLE 5:** Prognostic value of the key specific DEGs in Basal subtype, Her2 subtype, LumB subtype and total breast cancers.

**Basal**	**RFS**		**OS**	
Gene	HR (95% CI for HR)	**P**	HR (95% CI for HR)	**P**
SOX11	1.65 (1.25–2.16)	0.00031	1.75 (1.03–2.98)	0.0235
PLK1	0.82 (0.63–1.08)	0.15	0.56 (0.34–0.91)	0.019
BUB1	0.69 (0.53–0.89)	0.0045	0.44 (0.26–0.73)	0.00096
OGN	0.67 (0.49–0.93)	0.015	1.48 (0.76–2.86)	0.24
COL4A6	0.73 (0.56–0.95)	0.018	1.6 (0.98–2.64)	0.06
AGTR1	0.63 (0.48–0.82)	0.00048	0.79 (0.48–1.29)	0.34
ADRB2	0.56 (0.43–0.74)	2e-05	1.49 (0.91–2.46)	0.11
**BRCA**	**RFS**		**OS**	
Gene	HR (95% CI for HR)	**P**	HR (95% CI for HR)	**P**
SOX11	1.61 (1.44–1.8)	<1e-16	1.71 (1.37–2.13)	1.2e-06
PLK1	1.51 (1.34–1.7)	4.5e-12	1.56 (1.25–1.94)	6.7e-05
BUB1	1.84 (1.64–2.06)	<1e-16	2.12 (1.64–2.74)	4e-09
OGN	0.64 (0.55–0.75)	4e-08	0.57 (0.41–0.8)	0.0011
COL4A6	0.58 (0.52–0.65)	<1e-16	0.71 (0.55–0.93)	0.012
AGTR1	0.61 (0.54–0.68)	<1e-16	0.5 (0.39–0.65)	1.1e-07
ADRB2	0.59 (0.52–0.66)	<1e-16	0.62 (0.49–0.77)	2.4e-05
**HER2**	**RFS**		**OS**	
Gene	HR (95% CI for HR)	**P**	HR (95% CI for HR)	**P**
IL21R	0.42 (0.26–0.69)	0.00044	0.34 (0.15–0.79)	0.0083
IFI30	0.61 (0.42–0.9)	0.012	0.47 (0.24–0.9)	0.019
PI15	0.57 (0.38–0.83)	0.0034	1.77 (0.91–3.45)	0.087
FAM189A2	0.45 (0.31–0.67)	4.1e-05	0.63 (0.33–1.21)	0.16
MYZAP	1.55 (0.88–2.73)	0.13	5.79 (1.37–24.6)	0.007
**BRCA**	**RFS**		**OS**	
Gene	HR (95% CI for HR)	**P**	HR (95% CI for HR)	**P**
IL21R	0.67 (0.57–0.79)	1.4e-06	0.58 (0.39–0.86)	0.006
IFI30	1.37 (1.2–1.56)	1.8e-06	1.29 (1.04–1.6)	0.021
PI15	0.61 (0.55–0.68)	<1e-16	0.7 (0.56–0.88)	0.002
FAM189A2	0.62 (0.56–0.7)	1.9e-15	0.71 (0r.56–0.89)	0.0026
MYZAP	0.64 (0.54–0.74)	7.8e-09	0.68 (0.47–0.97)	0.03
**LumA**	**RFS**		**OS**	
Gene	HR (95% CI for HR)	**P**	HR (95% CI for HR)	**P**
SMIM22	1.3 (0.98–1.72)	0.073	1.44 (0.97–2.14)	0.067
**BRCA**	**RFS**		**OS**	
Gene	HR (95% CI for HR)	**P**	HR (95% CI for HR)	**P**
SMIM22	0.86 (0.73–1)	0.054	1.44 (0.97–2.14)	0.067
**LumB**	**RFS**		**OS**	
CNTD2	0.64 (0.53–0.78)	4.8e-06	0.85 (0.58–1.23)	0.38
NEURL	0.68 (0.56–0.83)	0.00011	1.26 (0.81–1.93)	0.3
STAC2	0.6 (0.43–0.85)	0.0039	0.39 (0.15–1.01)	0.044
AKR1C2	0.75 (0.54–1.04)	0.088	2.02 (1.03–3.98)	0.038
IL6	0.64 (0.53–0.78)	6.3e-06	0.79 (0.55–1.16)	0.23
FREM1	0.44 (0.32–0.61)	3.7e-07	0.42 (0.19–0.92)	0.026
HOXA4	0.71 (0.58–0.86)	0.00049	0.8 (0.55–1.17	0.25
**BRCA**	**RFS**		**OS**	
CNTD2	0.6 (0.53–0.67)	<1e-16	0.75 (0.59–0.94)	0.013
NEURL	0.62 (0.56–0.7)	<1e-16	0.84 (0.67–1.04)	0.11
STAC2	0.85 (0.72–1)	0.055	1.47 (1.04–2.07)	0.027
AKR1C2	0.75 (0.64–0.89)	0.00074	1.59 (1.16–2.2)	0.004
IL6	0.77 (0.69–0.86)	3.3e-06	0.86 (0.69–1.07)	0.17
FREM1	0.42 (0.36–0.49)	<1e-16	0.54 (0.38–0.78)	0.00068
HOXA4	0.64 (0.57–0.72)	2.4e-14	0.73 (0.58–0.91)	0.0055

For the key specific DEGs in the Her2 subtype, high expression of *PI15* and *FAM189A2* mRNA predicted high RFS respectively (*p* <0.05) (Table 5; Supplementary Figure S7A). Low expression of *MYZAP* mRNA predicted high OS (*p* < 0.05), and high expression of *IL21R* and *IFI30* mRNA predicted high RFS and OS respectively (*p* <0.05) (Table 5; Supplementary Figure S7A). The expression of *SPOCD1*, *JPH3*, *SAMD11*, *ATRNL1,* and *TNNI3K* had no significant correlation with RFS and OS of patients (*p* > 0.05).

For the key specific DEGs in the LumB subtype, high expression of *STAC2* and *FREM1* predicted high RFS and OS respectively (*p* <0.05, Table 5; Supplementary Figure S7B). High expression of *CNTD2*, *NEURL1*, *IL6,* and *HOXA4* predicted high RFS respectively (*p* <0.05), and low expression of *AKR1C2* predicted high OS (*p* < 0.05) (Table 5; Supplementary Figures S7B). The expression of *SYCE3*, *PPP1R1A,* and *HRCT1* had no significant correlation with RFS and OS of patients (*p* >0.05).

In addition, the prognostic value of the selected key common and specific DEGs was also analyzed in all breast cancer (BRCA) and shown in Table 4 and Table 5.

## Discussion

Accurate disease diagnosis, prognostic evaluation, and effective treatment are effective methods for reducing breast cancer mortality. Identification of the molecular subtypes and the molecular characteristics predicted by the subtypes through gene expression profiling can yield better understanding of the heterogeneity of breast cancer, enable the development of targeted treatment methods, and ultimately improve survival time. In the present study, we analyzed the mRNA expression data of breast cancer samples in TCGA database and identified 38 key common and specific DEGs in the five breast cancer subtypes, including the four overexpressed common DEGs and 34 specific DEGs with 17 genes upregulated and 17 genes downregulated. In order to better understand these DEGs, KEGG pathway and GO function were analyzed. The results of functional enrichment analysis showed that the significant DEGs were related to pathways such as systemic development, amino acid metabolism and cell cycle in BRCA. The regulation of the cell cycle is a hot issue and important content in life science research. Importantly, some DEGs have been validated and found to be associated with prognosis, which indicates that these genes not only control important pathways such as cellular processes, but also have high value in clinical diagnosis.

Endonuclease VIII-like 3 (NEIL3) is a DNA glycosylase protein involved in oxidative and DNA interstrand crosslink damage repair (Kazuya et al., 2016). *NEIL3*-overexpressing tumors accumulate mutations and chromosomal variations. *NEIL3* may be a potential prognostic marker for high-risk patients or an attractive therapeutic target for some cancers. CDC25C (cell division cycle 25C) may be a potential target for aspirin for inhibiting the proliferation of human breast cancer cells, and its gene function is mainly enriched in cell cycle and cell division (Zhu et al., 2019). NEK2 (never in mitosis gene A-related kinase 2) promotes tumor development through the Wnt signaling pathway, and may be a potential target for cancer treatment (Cappello et al., 2014; Wen et al., 2016; Tang et al., 2018). HCN2 (hyperpolarization-activated cyclic nucleotide-gated 2) plays an important role in neuronal excitability (Wu et al., 2018; Ramírez et al., 2018), whereas its role in tumors is unclear. According to the existing literature, except for NEK2 the expression and prognostic significance of the *NEIL3*, *CDC25C,* and *HCN2* genes are still unknown in breast cancer. Here, we found that low expression of *NEIL3*, *CDC25C,* and *NEK2* predicted high RFS and OS respectively (*p* < 0.05), and low expression of *HCN2* predicted high OS (*p* < 0.05) in all breast cancer. These results suggest that *NEIL3*, *CDC25C,* and *NEK2* may be involved in breast cancer recurrence. Further survival analysis of the four common DEGs in each subtype revealed that low expression of *NEK2* in the Basal-like subtype was associated with good RFS. Low expression of *NEIL3*, *CDC25C,* and *NEK2* in the LumA subtype was associated with good RFS respectively. Low expression of *NEK2* in the LumB subtype was associated with good RFS. These analytical data suggest that *NEK2* may be involved in the recurrence of Basal-, LumA- and LumB-subtype breast cancer, while *NEIL3* and *CDC25C* may only be involved in recurrence of LumA-subtype breast cancer. Functional studies are required to further determine their roles the recurrence of breast cancer in the future. In addition, to some extent these four key common DEGs can also be used as prognostic markers for breast cancer.

In the selected key specific DEGs for Basal-subtype breast cancer, the altered expression of SOX11, PLK1, BUB1, OGN, COL4A6, AGTR1, and ADRB2 were significantly correlated with the prognostic survival of the patients. SOX11 is a key regulator of proliferation and migration of Basal-subtype breast cancer cells, and is associated with poor prognosis (Shepherd et al., 2016). Our results show that SOX11 was specifically downregulated in Basal-subtype breast cancer. SOX11 high expression in the Basal-like subtype was associated with poor prognosis (RFS and OS), which was consistent with the prognostic analysis results in all breast cancer. These findings consistently suggest that SOX11 may play a carcinogenic role in the pathogenesis and development of Basal-subtype breast cancer. Further experimental studies are required to determine its precise functions in breast cancer in the future.

PLK1 plays key roles in the mitotic regulation of triple-negative breast cancer (TNBC) cells, and is associated with better prognosis in wild-type p53 breast tumors (King et al., 2012; de Cárcer et al., 2018; Ueda et al., 2019). Our results show that *PLK1* was specifically highly expressed in Basal-subtype breast cancer, and was mainly enriched in the cell cycle, mitosis and chromosome segregation pathways. *PLK1* does not like in all breast cancer that low expression predicted high RFS and OS (*p* < 0.05), *PLK1* overexpression improved prognosis in Basal-subtype breast cancer (*p* < 0.05). These data show that PLK1 may be a different important participant and a promising therapeutic target in Basal-subtype breast cancer.

BUB1 (budding uninhibited by benzimidazoles 1) plays a key role in the proliferation and radioresistance of glioblastoma (GBM) in a FOXM1-dependent manner (Yu et al., 2019). *BUB1* is overexpressed in breast cancer and is associated with poor clinical prognosis (Takagi et al., 2013). Our analysis revealed that *BUB1* was specifically highly expressed in the Basal-like subtype. However, high expression of *BUB1* was associated with good prognosis (RFS and OS) in Basal-subtype breast cancer, while low expression of *BUB1* predicted high RFS and OS (*p* < 0.05) in all breast cancer. This may be due to the different roles of *BUB1* in Basal-subtype and all breast cancer, which suggest a specific therapeutic target for Basal-subtype breast cancer. Moreover, whether BUB1 has a tumor suppressive activity remains uncertain in Basal-subtype breast cancer, and the mechanism of BUB1 also remains to be further explored. In addition, our results also show that *FOXM1* is specifically upregulated in Basal-subtype, and whether *BUB1* depends on *FOXM1* to play roles requires further exploration in Basal-subtype breast cancer.

Reduced *OGN* (osteoglycin) expression has been found in various types of cancers compared with normal tissues, and higher *OGN* expression is an indicator of increased survival and reduced cancer recurrence (Lee et al., 2003; Lomnytska et al., 2010). *OGN* can inhibit breast cancer cell proliferation (Xu et al., 2019). Our results found that *OGN* expression was significantly different in the Basal-like subtype from other breast cancer subtypes, and its expression in Basal-like subtype was lower than that in other subtypes. Low *OGN* expression was significantly associated with poor RFS in Basal-subtype breast cancer. These data suggest that *OGN* may have a different role and clinical value in Basal-like subtype. Further experimental studies are required to determine its precise functions and clinical value in Basal-subtype breast cancer in the future.

COL4A6 (collagen type IV alpha six chain) is involved in cancer progression and invasion, whose expression correlates positively with the DFS of patients in prostate cancer (Ma et al., 2020). *COL4A6* was also identified as a key gene associated with survival of cancer cells in breast cancer (Li et al., 2020). Our results show that *COL4A6* was significantly downregulated in patients with Basal-subtype breast cancer, and this low expression was associated with poor prognosis (RFS). AGTR1 (angiotensin II receptor type 1) is associated with tumor growth, tumor metastasis and drug resistance (Pu et al., 2017; Zhang et al., 2019a; Ma et al., 2019). Studies have also shown that *AGTR1* may be a potential therapeutic target in early breast cancer with lymph node metastasis (Ma et al., 2019). *AGTR1* is overexpressed in LumA- and LumB-subtype breast cancer, which is associated with aggressive features and decreased OS (Ekambaram et al., 2018). Our results show that *AGTR1* was significantly downregulated in the Basal-like subtype, and this low expression was associated with poor prognosis (RFS). ADRB2 (adrenoceptor beta 2) plays an important role in the progression and metastasis of various tumors (Zhang et al., 2019b; Kulik, 2019). ADRB2 single-nucleotide polymorphisms (SNPs) rs1042713 and rs1042714 may influence the response to β blockers in breast cancer treatment (Xie et al., 2019). Here, *ADRB2* was found to be specifically downregulated in basal-subtype breast cancer, and this low *ADRB2* expression was associated with poor prognosis (RFS) in Basal-subtype breast cancer. These data indicate that *COL4A6, AGTR1,* and *ADRB2* may be involved in the recurrence of Basal-subtype breast cancer, and further studies are required to determine their precise roles in Basal-subtype breast cancer.

In brief, the analytical data suggest that *SOX11*, *PLK1,* and *BUB1* may be involved in tumorigenesis to improve OS of patients with Basal-subtype breast cancer. SOX11, PLK1, BUB1, OGN, COL4A6, AGTR1, and ADRB2 may be involved in the recurrence of Basal-subtype breast cancer, and to some extent the PLK1, BUB1, OGN, COL4A6, AGTR1, and ADRB2 can be used as the specific prognostic markers for Basal-subtype breast cancer.

In the selected key specific DEGs for Her2-subtype breast cancer, the expression of *IL21R, IFI30, PI15, FAM189A2,* and *MYZAP* were significantly correlated with the prognostic survival of the patients. *IL21R* (interleukin 21 receptor) knock-down may sensitize cells to anti-tumor therapy by targeting MMPs, and participate in tumor progression and metastasis in advanced breast cancer (Kim et al., 2014; Wang et al., 2015). In our analysis, *IL21R* was specifically upregulated, and this high expression predicted high RFS and OS (*p* < 0.05) in Her2-subtype breast cancer, which was consistent with the prognostic analysis results in all breast cancer.

*IFI30* is highly expressed in glioma and associated with chemotherapy response (Zhu et al., 2020). Our analysis showed that *IFI30* were specifically upregulated and this high expression predicted high RFS and OS (*p* < 0.05) in Her2-subtype breast cancer, while high *IFI30* expression predicted low RFS and OS (*p* < 0.05) in all breast cancer. These data suggest that *IFI30* may have a different role and clinical value in Her2-subtype. Further studies are required to determine the specific role and clinical value as well as the mechanism in Her2-subtype breast cancer.

*PI15* has been identified as a potential diagnostic marker in colorectal carcinoma and cholangiocarcinoma (Tuupanen et al., 2014; Jiang et al., 2019). Our results showed that *PI15* was specifically downregulated in the Her2 subtype, and this high expression predicted high RFS in Her2-subtype breast cancer (*p* < 0.05). Thus, PI15 may be also a potential prognostic factor in Her2-subtype breast cancer.

*FAM189A2* is a potential therapeutic target, and its low expression is associated with poor prognosis in oral squamous cell carcinoma (Hu et al., 2020). The role of MYZAP (myocardium-enriched zonula occludens-1-associated protein) in tumors is completely unclear at present. Our analysis showed that *FAM189A2* and *MYZAP* were specifically downregulated in the Her2 subtype, and high *FAM189A2* expression predicted high RFS in Her2-subtype breast cancer (*p* < 0.05), while low *MYZAP* expression predicted high OS in Her2-subtype breast cancer (*p* < 0.05). These analysis data show that *FAM189A2* and *MYZAP* may be potential prognostic factors in Her2-subtype breast cancer, and their main roles are required to further study.

In brief, the data in Her2-subtype breast cancer suggest that *IL21R*, *IFI30,* and *MYZAP* may be involved in the tumorigenesis to improve OS of patients with Her2-subtype breast cancer. *IL21R, IFI30, PI15,* and *FAM189A2* may be involved in recurrence of Her2-subtype breast cancer *IFI30, PI15, FAM189A2,* and *MYZAP* can be used as the specific prognostic markers for Her2-subtype breast cancer.

In the selected key specific DEGs for LumB-subtype breast cancer, the expression of *CNTD2, NEURL, STAC2, AKR1C2, IL6*, *FREM1,* and *HOXA4* were significantly correlated with the prognostic survival of the patients. *CNTD2* can promote colon and lung cancer cell proliferation and migration (Gasa et al., 2017; Abril et al., 2018). Here, we found that *CNTD2* was specifically upregulated and the high expression predicted high RFS in LumB-subtype breast cancer (*p* < 0.05), and high *CNTD2* expression predicted high RFS (*p* < 0.05) and OS (*p* < 0.05) in all breast cancer. *NEURL1* is a potential suppressor of multiple tumors, and its low expression is associated with reduced metastasis-free survival (Teider et al., 2010; Kenawy et al., 2019). Our results show that *NEURL1* was specifically upregulated, and the high expression predicted high RFS in LumB-subtype breast cancer (*p* < 0.05), which is consistent with the prognostic conclusion of all breast cancer. These results revealed that *CNTD2* and *NEURL1* may play similar roles and prognostic value in breast cancer and LumB-subtype breast cancer.

STAC2 belongs to a small family of SH3 and cysteine-rich adaptor proteins and is expressed in various tissue types (Nelson et al., 2013). FREM1 is expressed in the developing skin epidermis and in many differentiated epidermal structures (Smyth et al., 2004). FREM1 may be a potential candidate for immunotherapy targets in breast cancer and may be used as a prognostic marker for DFS (Zhang et al., 2020). However, the roles and mechanisms of STAC2 and FREM1 in tumors are still largerly unclear. Our results showed that *STAC2* and *FREM1* were specifically downregulated and their low expressions predicted low RFS and OS (*p* < 0.05) in LumB-subtype breast cancer. The prognostic result of FREM1 is consistent with that in all breast cancer, while low *STAC2* expression predicted high OS (*p* < 0.05) in all breast cancer. Thus, *FREM1* may play the similar roles and prognostic values, whereas *STAC2* may play different roles and prognostic values in breast cancer and LumB-subtype breast cancer. Further studies are needed to determine the exact roles prognostic values of STAC2 and FREM1 in breast cancer and LumB-subtype breast cancer.

*AKR1C2* has an inhibitory effect in the development of squamous cell carcinoma and breast cancer (Li et al., 2016b; Wenners et al., 2016). However, it is a positive regulator in promoting liver cancer metastasis (Li et al., 2016a). We found that *AKR1C2* was specifically downregulated, and the low expression predicted increased OS in LumB-subtype breast cancer. Therefore, *AKR1C2* may play an important role in LumB-subtype breast cancer. Further studies are required to determine the function of AKR1C2 in LumB-subtype breast cancer.

High *IL6* expression can eliminate the anti-tumor effect of the inhibitor RO4929097 in lung cancer and glioma (He et al., 2011). *HOXA4* is overexpressed in colorectal cancer and epithelial ovarian cancer (Bhatlekar et al., 2014), and its overexpression can inhibit cancer cell growth and invasion which is associated with Wnt pathway in lung cancer (Cheng et al., 2018). In breast cancer, HOXA4 exhibits increased DNA methylation and decreased gene expression (Li et al., 2019). Here, *IL6* and *HOXA4* were found to be specifically downregulated, and their low expression predicted low RFS in LumB-subtype breast cancer (*p* < 0.05), and the prognostic analysis result of *IL6* was consistent with tha in all breast cancer. The data suggested that *IL6* and *HOXA4* may play key roles in LumB-subtype breast cancer.

In general, the analytical data suggest that *STAC2*, *FREM1,* and *AKR1C2* may be involved in the tumorigenesis to improve OS of patients with LumB-subtype breast cancer. *CNTD2, NEURL, STAC2, IL6*, *FREM1,* and HOXA4 may be involved in recurrence of LumB-subtype breast cancer. Moreover, *STAC2, AKR1C2,* and *HOXA4* can be used as the specific prognostic markers for LumB-subtype breast cancer.

In addition, the independent validation reveal that the expressions of the selected key specific DEGs are highly consistent with TCGA data, whereas the expressions of the selected key common DEGs seem to be very different in the independent BRCA samples of GSE65216 database, which may explain the relatively consistent prognostic values for key specific DEGs in each subtype but very different prognostic values for the common DEGs in different subtypes to some extent.

The poor prognosis of the different breast cancer subtypes is mainly due to the lack of effective therapeutic targets. Therefore, finding new therapeutic targets for improving the subtypes’ prognosis is essential. We believe that these key genes may be potential markers for the different breast cancer subtypes. Although these findings have great potential value, our study still has some limitations. The specific prognostic values of these genes need to be verified in independent large cohorts. The precise roles and mechanisms of the candidate genes are required to explore by experimental studies to enhance the molecular basis of these genes in the future clinical application. In summary, our findings may provide new insights into the characteristics of each subtype of breast cancer and provide potential new therapeutic targets for different subtypes in the future.

## Data Availability

Publicly available datasets were analyzed in this study. This data can be found here: cBioPortal at https://identifiers.org/cbioportal:brca_metabric (METABRIC data) and at https://xena.ucsc.edu/public/ (TCGA data).
