# Supplementary material for: Screening and Identification of Key Common and Specific Genes and Their Prognostic Roles in Different Molecular Subtypes of Breast Cancer
Source: Front Mol Biosci. 2021 Feb 11;8:619110. doi: 10.3389/fmolb.2021.619110 (PMC7905399; doi:10.3389/fmolb.2021.619110)
Supplement: Supplementary file 1 [file table1.docx]

Table.1 Number of co-expressed and specific DEGs in the breast cancer subtypes.

| PAM50 subtype | Common | Basal-like | Her2 | LumA | LumB | Normal-like |
| --- | --- | --- | --- | --- | --- | --- |
| UP | 4 | 25 | 34 | 5 | 9 | 3 |
| DOWN | 0 | 55 | 21 | 0 | 47 | 0 |

Table.2 Genes with consistent expression with TCGA screening results were verified via the METABRIC database and BC-GenExMiner v4.5.

| PAM50 subtype | Up Gene name | Down Gene name |
| --- | --- | --- |
| Basal-Her2-LumA-LumB-Normal-like | NEIL3/CDC25C/NEK2/HCN2 | / |
| Basal | SLCO5A1/GPR19/LCTL/MCM10/HPDL/SOX11/RAD54L/CDCA2/MSLN/GTSE1/NDC80/BUB1/COL22A1/KIF2C/PLK1/FOXM1/CDCA3/NCAPG/AUNIP/MIR4292/CCNE1 | LINC00504/TFF3/CYP4B1/TTC36/ DYNLRB2/OGN/ ACSM5/MS4A2/MIR4697HG/C16orf89/PTPRT/ MYRIP/CAPN8/AGTR1/ PFKFB1/PGR/ COL4A6/ABCC11/ DHRS2/NDNF/PLIN5/FER1L5/AZGP1P1/GRIK3/NEK10/RANBP3L/FAM198B-AS1/GFRA1/SOWAHA/CLEC3B/LRRN3/FAM162B/AK5/IQUB/SEMA3E/ ADRB2/GLDN/DACH1/LIPE/ LOC105371730 |
| Her2 | LOC105372233/SPOCD1/ IL21R/JPH3/SAMD11/ IFI30/JSRP1/ANGPTL6/IL4I1/VSTM2L/ MMP3/HES6/S100A8/ASCL2/TCHH/NXPH4/LBX2/ LOC105371849/SIX2 | LINC00173/ATRNL1/TNNI3K/PI15/FAM189A2/LOC100505635/MYZAP/SLC4A4/LRP2/NAT8L/OXTR/SLC25A27/ FGF14-AS2/LINC00639/TRMT9B |
| LumA | MISP/SMIM22 | / |
| LumB | CNTD2/NEURL1/SYCE3 | STAC2/PPP1R1A/HRCT1/AKR1C2/IL6/FREM1/HOXA4/TMOD1/PTGS2/PTGS2/FAM107A/IL33/TRIM29/TMEM220-AS1/PROX1/GFRA2/SLC28A3/HCAR2/PPARGC1A/GNG12-AS1/TDRD10/SOX8/TP63/TTYH1/PTH2R/ SLC2A12/KCNA6/PROM1/KRT17/ C2orf88/PAMR1/PPP1R14A / BCL11A |
| Normal-like | IDH1-AS1/TMEM233 | / |

(Genes annotated in yellow are those for which validation results were discordant or no data were available in the METABRIC database and BC genexminer v4.5)

Table.3 The key common and specific DEGs selected were verified in the GSE65216 dataset.

| **Basal-like** | | |
| --- | --- | --- |
| **Gene name** | **TCGA** | **GSE65216** |
| NEIL3 | Up | No change |
| HCN2 | Up | Down |
| NEK2 | Up | Up |
| CDC25C | Up | Up |
| SOX11 | Up | Up |
| PLK1 | Up | Up |
| BUB1 | Up | Up |
| OGN | Down | Down |
| AGTR1 | Down | Down |
| COL4A6 | Down | Down |
| ADRB2 | Down | Down |
| DYNLRB2 | Down | Down |
| HPDL | Up | Up |
| MCM10 | Up | Up |
| **Her2** | | |
| **Gene name** | **TCGA** | **GSE65216** |
| NEIL3 | Up | Down |
| HCN2 | Up | Down |
| NEK2 | Up | No change |
| CDC25C | Up | Down |
| ATRNL1 | Down | Down |
| FAM189A2 | Down | Down |
| IFI30 | Up | Up |
| IL21R | Up | No change |
| JPH3 | Up | Down |
| MYZAP | Down | Down |
| PI15 | Down | Down |
| SAMD11 | Up | Up |
| SPOCD1 | Up | No change |
| TNNI3K | Down | No change |
| **LumA** | | |
| **Gene name** | **TCGA** | **GSE65216** |
| NEIL3 | Up | No change |
| HCN2 | Up | Down |
| NEK2 | Up | No change |
| CDC25C | Up | No change |
| MISP | Up | Up |
| SMIM2 | Up | Up |
| **LumB** | | |
| **Gene name** | **TCGA** | **GSE65216** |
| NEIL3 | Up | No change |
| HCN2 | Up | Down |
| NEK2 | Up | Down |
| CDC25C | Up | Down |
| AKR1C2 | Down | Down |
| CNTD2 | Up | No change |
| FREM1 | Down | Down |
| HOXA4 | Down | Down |
| HRCT1 | Down | Down |
| IL6 | Down | No change |
| NEURL1 | Up | Down |
| PPP1R1A | Down | Down |
| STAC2 | Down | Down |
| SYCE3 | Up | No change |

Table. 4 Prognostic value of the key common DEGs in total and different subtype breast cancers

| **Basal** | **RFS** | | **OS** | |
| --- | --- | --- | --- | --- |
| Gene | HR(95% CI for HR) | P | HR(95% CI for HR) | P |
| NEIL3 | 0.68(0.53-0.88) | 0.003 | 0.57(0.34-0.93) | 0.022 |
| CDC25C | 0.91(0.7-1.17) | 0.45 | 0.5(0.3-0.83) | 0.0063 |
| NEK2 | 1.35(1.03-1.79) | 0.031 | 0.55(0.34-0.9) | 0.016 |
| HCN2 | 0.77(0.58-1.03) | 0.074 | 1.36(0.83-2.23) | 0.23 |
| **HER2** | **RFS** | | **OS** | |
| NEIL3 | 0.59(0.4-0.86) | 0.006 | 1.38(0.71-2.7) | 0.34 |
| CDC25C | 0.7(0.47-1.04) | 0.078 | 0.56(0.26-1.23) | 0.14 |
| NEK2 | 1.34(0.91-1.97) | 0.13 | 0.55(0.23-1.32) | 0.17 |
| HCN2 | 1.54(0.98-2.42) | 0.057 | 1.94(0.91-4.11) | 0.079 |
| **Lum A** | **RFS** | | **OS** | |
| NEIL3 | 1.29(1.08-1.54) | 0.0041 | 1.79(1.24-2.59) | 0.0017 |
| CDC25C | 1.64(1.37-1.96) | 6e-08 | 2(1.4-2.85) | 9.2e-05 |
| NEK2 | 2.2(1.85-2.6) | <1e-16 | 2.5(1.75-3.57) | 1.6e-07 |
| HCN2 | 0.86(0.72-1.02) | 0.09 | 1.5(1.03-2.19) | 0.034 |
| **Lum B** | **RFS** | | **OS** | |
| NEIL3 | 0.81(0.67-0.98) | 0.03 | 1.73(1.11-2.69) | 0.014 |
| CDC25C | 1.19(0.99-1.45) | 0.068 | 1.49(1.03-2.15) | 0.035 |
| NEK2 | 1.71(1.37-2.14) | 1.7e-06 | 1.86(1.17-2.96) | 0.0082 |
| HCN2 | 0.74(0.6-0.91) | 0.0047 | 1.36(0.94-1.97) | 0.1 |
| **BRCA** | **RFS** | | **OS** | |
| NEIL3 | 1.12(1.07-1.38) | 0.0022 | 1.63(1.27-2.11) | 0.00014 |
| CDC25C | 1.45(1.3-1.61) | 2.6e-11 | 1.54(1.24-1.91) | 8.9e-05 |
| NEK2 | 1.91(1.7-2.14) | <1e-16 | 2.08(1.62-2.67) | 4.8e-09 |
| HCN2 | 0.83(0.74-0.94) | 0.0033 | 1.39(1.1-1.77) | 0.0064 |

Table.5 Prognostic value of the key specific DEGs in Basal subtype, Her2 subtype, LumB subtype and total breast cancers.

| **Basal** | **RFS** | | **OS** | |
| --- | --- | --- | --- | --- |
| Gene | HR(95% CI for HR) | P | HR(95% CI for HR) | P |
| SOX11 | 1.65(1.25-2.16) | 0.00031 | 1.75(1.03-2.98) | 0.0235 |
| PLK1 | 0.82(0.63-1.08) | 0.15 | 0.56(0.34-0.91) | 0.019 |
| BUB1 | 0.69(0.53-0.89) | 0.0045 | 0.44(0.26-0.73) | 0.00096 |
| OGN | 0.67(0.49-0.93) | 0.015 | 1.48(0.76-2.86) | 0.24 |
| COL4A6 | 0.73(0.56-0.95) | 0.018 | 1.6(0.98-2.64) | 0.06 |
| AGTR1 | 0.63(0.48-0.82) | 0.00048 | 0.79(0.48-1.29) | 0.34 |
| ADRB2 | 0.56(0.43-0.74) | 2e-05 | 1.49(0.91-2.46) | 0.11 |
| **BRCA** | **RFS** | | **OS** | |
| Gene | HR(95% CI for HR) | P | HR(95% CI for HR) | P |
| SOX11 | 1.61(1.44-1.8) | <1e-16 | 1.71(1.37-2.13) | 1.2e-06 |
| PLK1 | 1.51(1.34-1.7) | 4.5e-12 | 1.56(1.25-1.94) | 6.7e-05 |
| BUB1 | 1.84(1.64-2.06) | <1e-16 | 2.12(1.64-2.74) | 4e-09 |
| OGN | 0.64(0.55-0.75) | 4e-08 | 0.57(0.41-0.8) | 0.0011 |
| COL4A6 | 0.58(0.52-0.65) | <1e-16 | 0.71(0.55-0.93) | 0.012 |
| AGTR1 | 0.61(0.54-0.68) | <1e-16 | 0.5(0.39-0.65) | 1.1e-07 |
| ADRB2 | 0.59(0.52-0.66) | <1e-16 | 0.62(0.49-0.77) | 2.4e-05 |
| **HER2** | **RFS** | | **OS** | |
| Gene | HR(95% CI for HR) | P | HR(95% CI for HR) | P |
| IL21R | 0.42(0.26-0.69) | 0.00044 | 0.34(0.15-0.79) | 0.0083 |
| IFI30 | 0.61(0.42-0.9) | 0.012 | 0.47(0.24-0.9) | 0.019 |
| PI15 | 0.57(0.38-0.83) | 0.0034 | 1.77(0.91-3.45) | 0.087 |
| FAM189A2 | 0.45(0.31-0.67) | 4.1e-05 | 0.63(0.33-1.21) | 0.16 |
| MYZAP | 1.55（0.88-2.73） | 0.13 | 5.79（1.37-24.6） | 0.007 |
| **BRCA** | **RFS** | | **OS** | |
| Gene | HR(95% CI for HR) | P | HR(95% CI for HR) | P |
| IL21R | 0.67(0.57-0.79) | 1.4e-06 | 0.58(0.39-0.86) | 0.006 |
| IFI30 | 1.37(1.2-1.56) | 1.8e-06 | 1.29(1.04-1.6) | 0.021 |
| PI15 | 0.61(0.55-0.68) | <1e-16 | 0.7(0.56-0.88) | 0.002 |
| FAM189A2 | 0.62(0.56-0.7) | 1.9e-15 | 0.71(0r.56-0.89) | 0.0026 |
| MYZAP | 0.64(0.54-0.74) | 7.8e-09 | 0.68(0.47-0.97) | 0.03 |
| LumA | **RFS** | | **OS** | |
| Gene | HR(95% CI for HR) | P | HR(95% CI for HR) | P |
| SMIM22 | 1.3(0.98-1.72) | 0.073 | 1.44(0.97-2.14) | 0.067 |
| **BRCA** | **RFS** | | **OS** | |
| Gene | HR(95% CI for HR) | P | HR(95% CI for HR) | P |
| SMIM22 | 0.86(0.73-1) | 0.054 | 1.44(0.97-2.14) | 0.067 |
| **LumB** | **RFS** | | **OS** | |
| CNTD2 | 0.64(0.53-0.78) | 4.8e-06 | 0.85(0.58-1.23) | 0.38 |
| NEURL | 0.68(0.56-0.83) | 0.00011 | 1.26(0.81-1.93) | 0.3 |
| STAC2 | 0.6(0.43-0.85) | 0.0039 | 0.39(0.15-1.01) | 0.044 |
| AKR1C2 | 0.75(0.54-1.04) | 0.088 | 2.02(1.03-3.98) | 0.038 |
| IL6 | 0.64(0.53-0.78) | 6.3e-06 | 0.79(0.55-1.16) | 0.23 |
| FREM1 | 0.44(0.32-0.61) | 3.7e-07 | 0.42(0.19-0.92) | 0.026 |
| HOXA4 | 0.71(0.58-0.86) | 0.00049 | 0.8(0.55-1.17 | 0.25 |
| **BRCA** | **RFS** | | **OS** | |
| CNTD2 | 0.6(0.53-0.67) | <1e-16 | 0.75(0.59-0.94) | 0.013 |
| NEURL | 0.62(0.56-0.7) | <1e-16 | 0.84(0.67-1.04) | 0.11 |
| STAC2 | 0.85(0.72-1) | 0.055 | 1.47(1.04-2.07) | 0.027 |
| AKR1C2 | 0.75(0.64-0.89) | 0.00074 | 1.59(1.16-2.2) | 0.004 |
| IL6 | 0.77(0.69-0.86) | 3.3e-06 | 0.86(0.69-1.07) | 0.17 |
| FREM1 | 0.42(0.36-0.49) | <1e-16 | 0.54(0.38-0.78) | 0.00068 |
| HOXA4 | 0.64(0.57-0.72) | 2.4e-14 | 0.73(0.58-0.91) | 0.0055 |
