# Supplementary figures and images for: Screening and Identification of Key Common and Specific Genes and Their Prognostic Roles in Different Molecular Subtypes of Breast Cancer
Source: Front Mol Biosci. 2021 Feb 11;8:619110. doi: 10.3389/fmolb.2021.619110 (PMC7905399; doi:10.3389/fmolb.2021.619110)

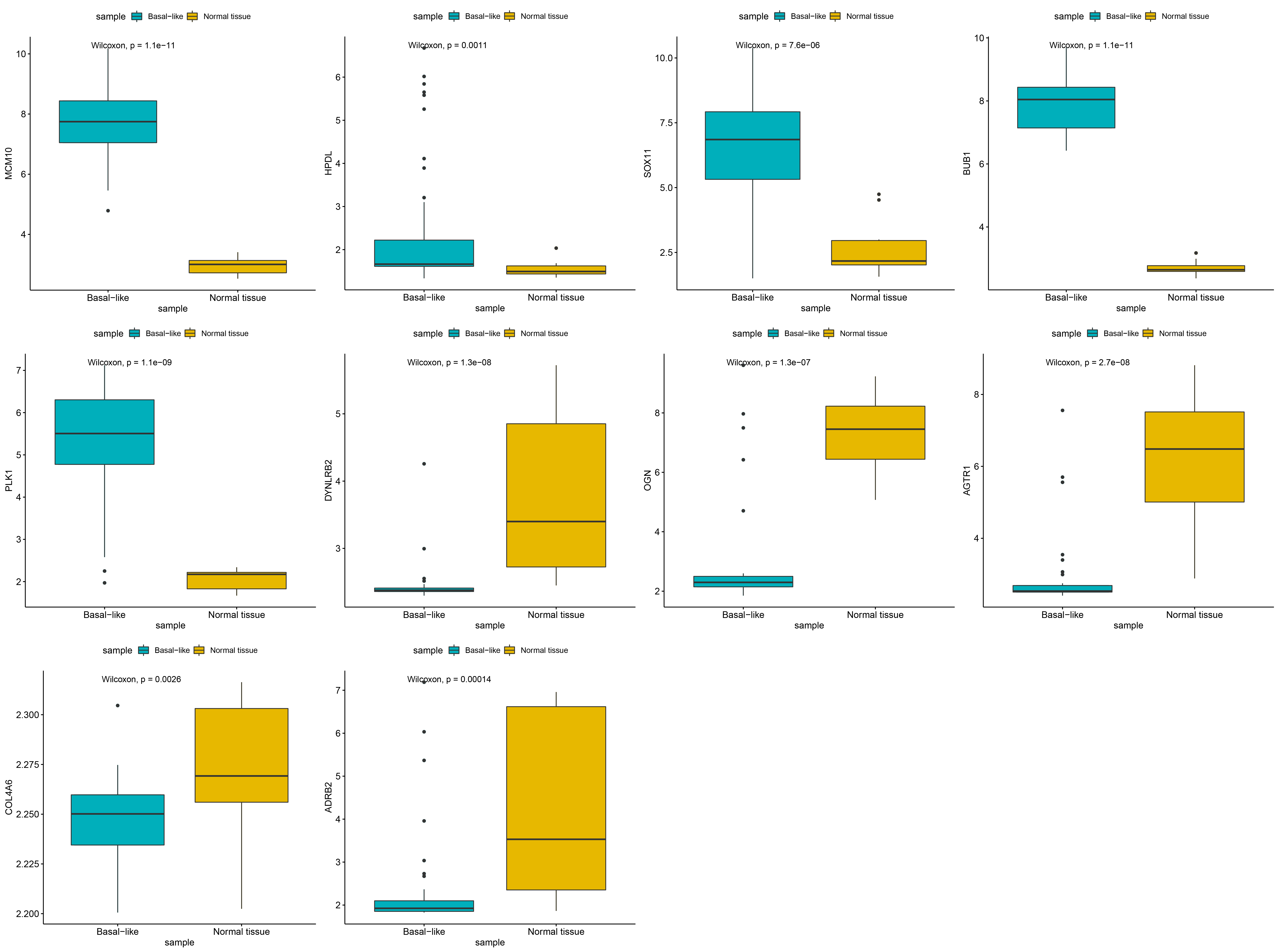

Supplement: Supplementary file 3 [file image1.tif]

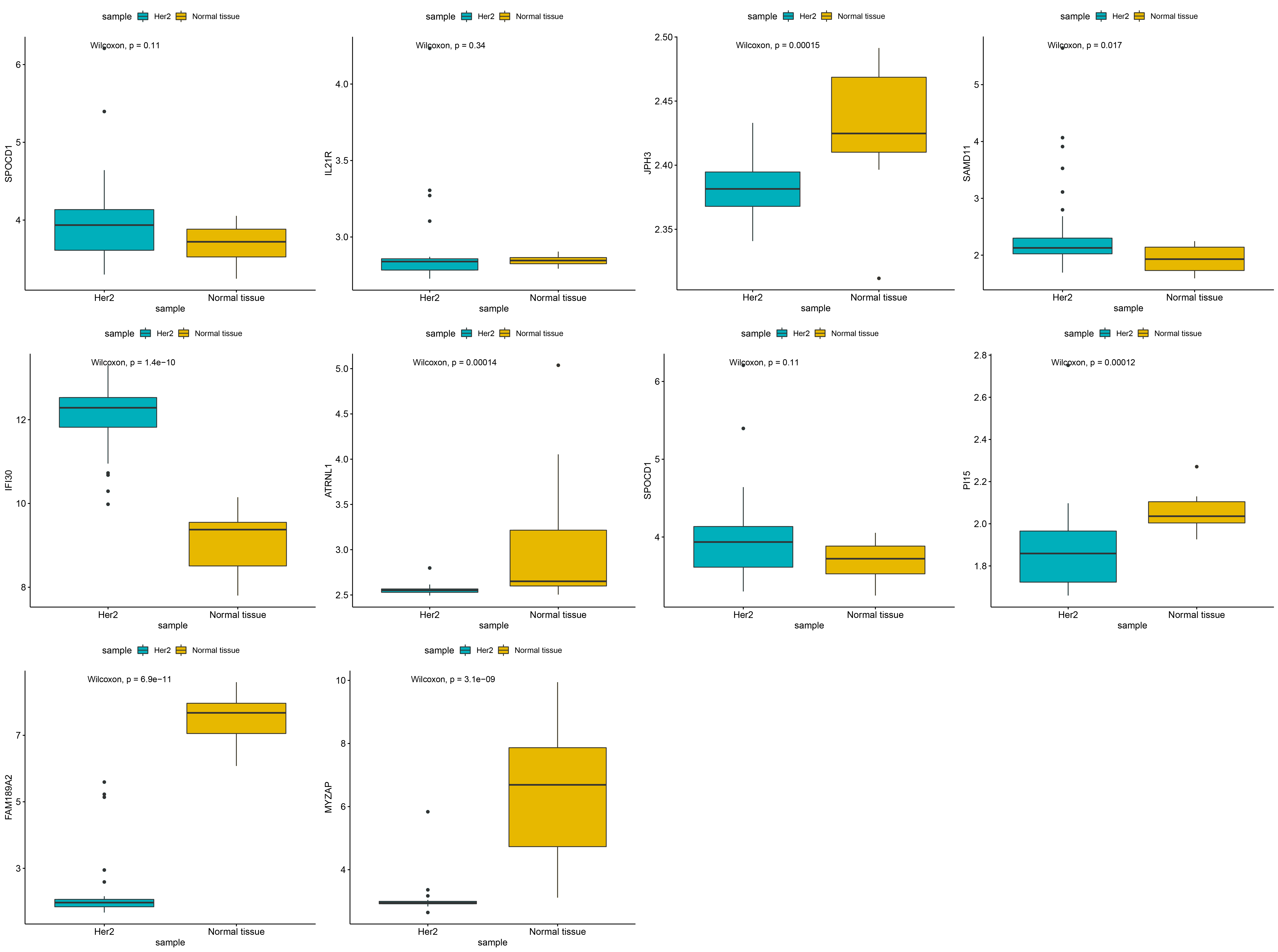

Supplement: Supplementary file 4 [file image2.tif]

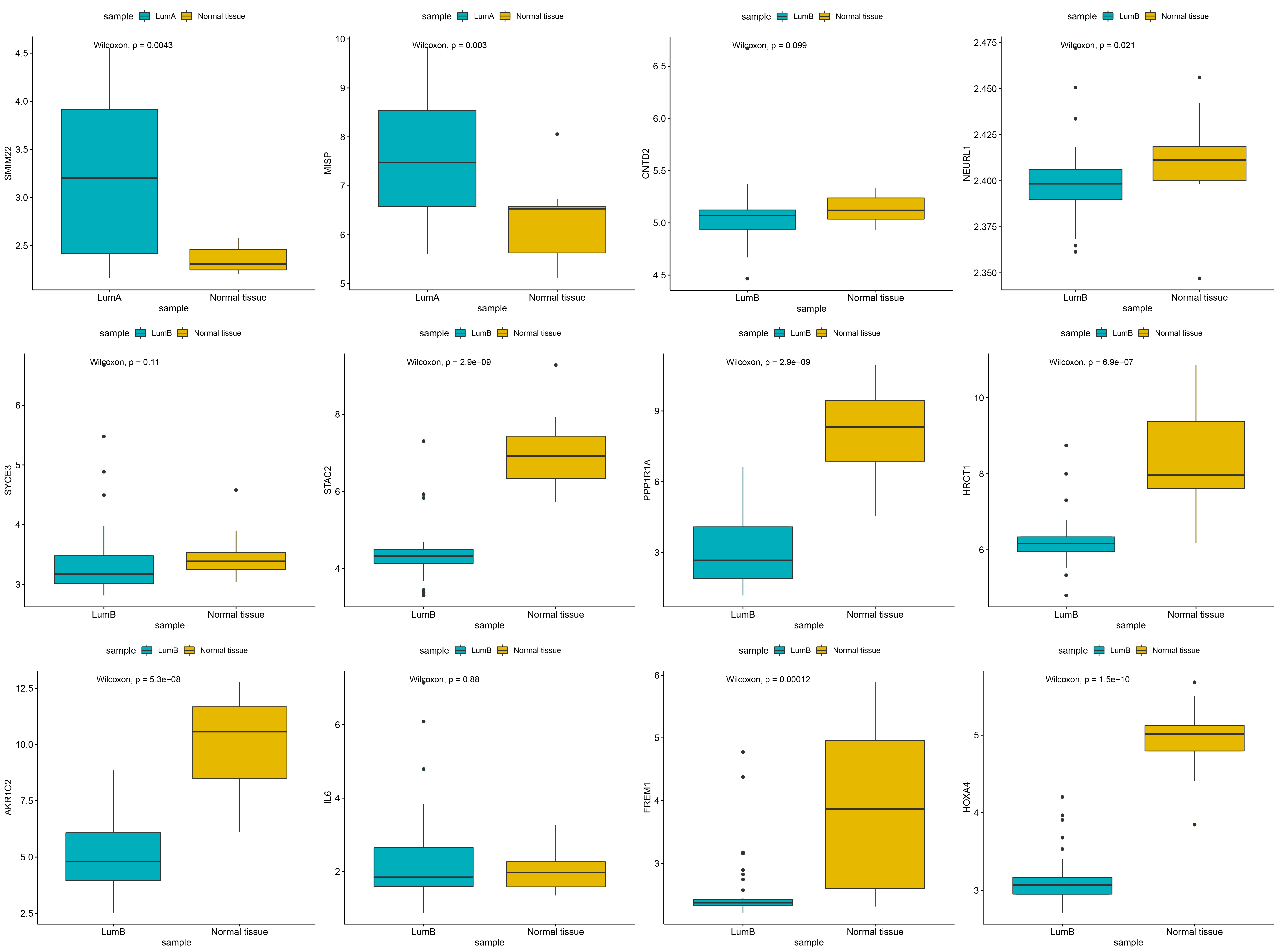

Supplement: Supplementary file 5 [file image3.tif]

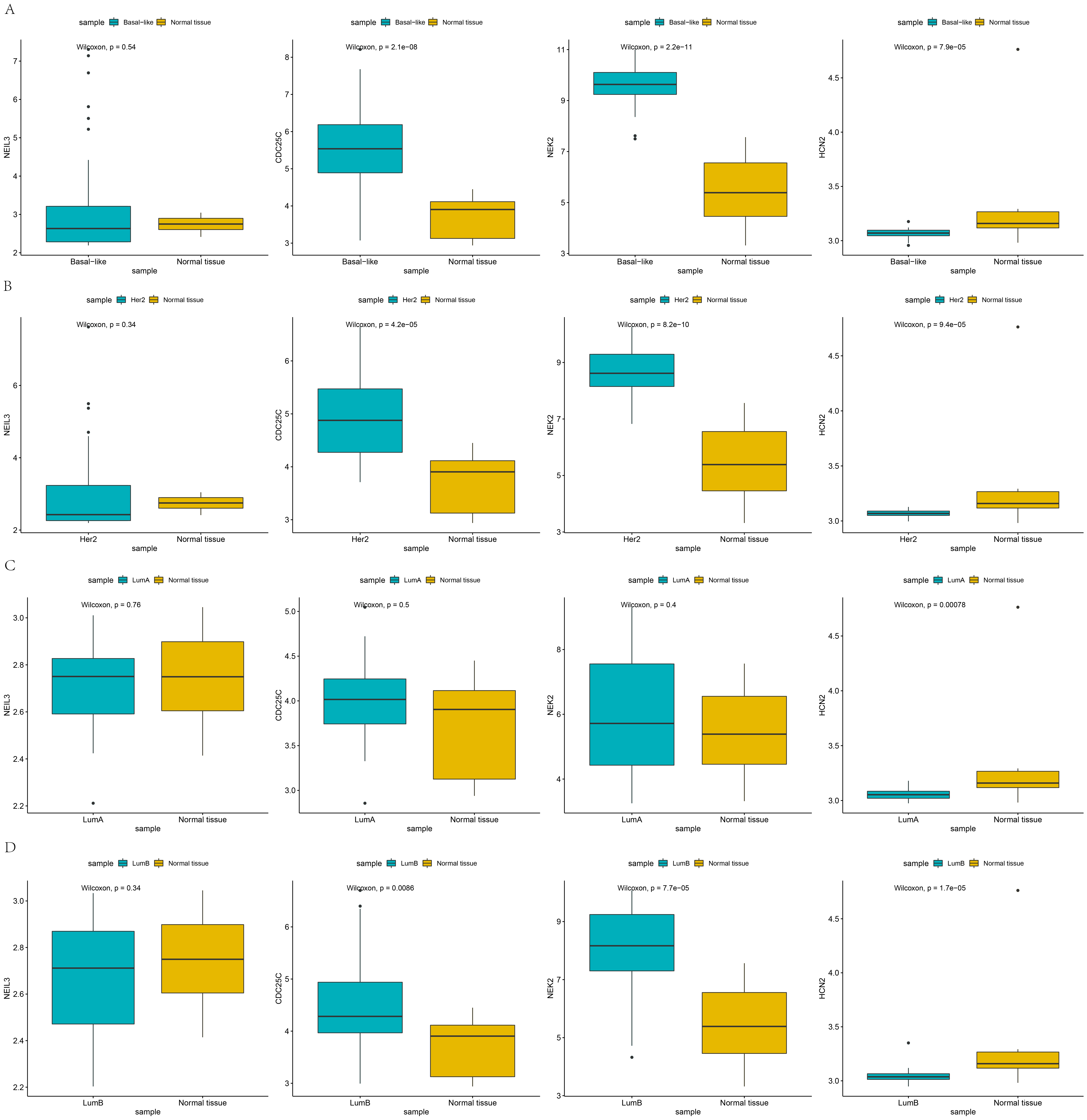

Supplement: Supplementary file 6 [file image4.tif]
